# Prospective Memory Performance in Non-Psychotic First-Degree Relatives of Patients with Schizophrenia: A Controlled Study

**DOI:** 10.1371/journal.pone.0111562

**Published:** 2014-11-03

**Authors:** Fu-Chun Zhou, Wei-Min Hou, Chuan-Yue Wang, Gabor S. Ungvari, Helen F. K. Chiu, Christoph U. Correll, David H. K. Shum, David Man, Deng-Tang Liu, Yu-Tao Xiang

**Affiliations:** 1 Beijing Key Laboratory of Mental Disorders, Beijing Anding Hospital, Capital Medical University, Beijing, China; 2 Beijing Daxing Mental Health Center, Beijing, China; 3 School of Psychiatry & Clinical Neurosciences, University of Western Australia, Perth, Australia; 4 Department of Psychiatry, Chinese University of Hong Kong, Hong Kong SAR, China; 5 Division of Psychiatry Research, The Zucker Hillside Hospital, North Shore-Long Island Jewish Health System, Glen Oaks, New York, United States of America; 6 School of Psychology and Griffith Health Institute, Griffith University, Brisbane, Queensland, Australia; 7 Department of Rehabilitation Sciences, Hong Kong Polytechnic University, Hong Kong SAR, China; 8 Department of Psychiatry, Shanghai Mental Health Center, Shanghai Jiao Tong University School of Medicine, Shanghai, China; 9 Faculty of Health Sciences, University of Macau, Macao SAR, China; Benito Menni Complejo Asistencial en Salud Mental, Spain

## Abstract

**Objective:**

We aimed at investigating prospective memory and its socio-demographic and neurocognitive correlates in non-psychotic, first-degree relatives (FDRs) of patients with schizophrenia compared to patients with first episode schizophrenia (FES), and healthy controls (HCs).

**Methods:**

Forty-seven FES patients, 50 non-psychotic FDRs (23 offspring and 27 siblings) of patients with chronic schizophrenia (unrelated to the FES group) and 51 HCs were studied. The Chinese version of the Cambridge Prospective Memory Test (C-CAMPROMPT) was used to measure time-based prospective memory (TBPM) and event-based prospective memory (EBPM) performance. Other cognitive functions (involving respective memory and executive functions) were evaluated with standardized tests.

**Results:**

After controlling for basic demographic characteristics including age, gender and educational level, there was a significant difference between FDRs, FES and HCs with respect to both TBPM (F_(2,142)_ = 10.4, p<0.001) and EBPM (F_(2,142)_ = 10.8, p<0.001). Multiple linear regression analyses revealed that lower scores of the Hopkins Verbal Learning Test-Revised (HVLT-R) and the STROOP Word-Color Test (SWCT) contributed to TBPM impairment, while lower educational level and higher scores of the Color Trails Test-2 (CTT-2) contributed to EBPM deficit in FDRs.

**Conclusions:**

FDRs share similar but attenuated prospective memory impairments with schizophrenia patients, suggesting that prospective memory deficits may represent an endophenotype of schizophrenia.

## Introduction

Schizophrenia is a frequently debilitating illness, manifesting primarily with positive and negative psychotic symptoms and cognitive impairment. While a wide range of neurocognitive deficits occur in schizophrenia, the impairments are especially prominent in psychomotor speed, memory, attention, reasoning, and social cognition. Memory impairment has been studied extensively [1], [2], [3]. Although previous studies focused more on retrospective memory (RM; the ability to remember past information), there is an increasing number of studies that addressed the impairment of prospective memory (PM) in schizophrenia.

PM, defined as “memory for activities to be performed in the future”, is usually subdivided into time-based PM (TBPM) and event-based PM (EBPM) according to the nature of the cue associated with the future intention. TBPM is the ability to remember carrying out an intention at a specific time in the future. EBPM is defined as the ability to remember performing an intended action when a certain cue appears [4]. PM has been suggested to contribute to the disorganized lifestyle in people with schizophrenia [5]. PM tasks involves the encoding of an intention, retaining the information, executing the intention, and evaluating the outcome [6]. PM involves both retrospective memory and an executive cognition component [7], allowing retrieval of the intended content and the specific action that must be executed [8].

PM deficits have been consistently found in both chronic and first episode schizophrenia [9], [10], [11], [12], [13], [14], [15], [16], [17]. A recent meta-analysis showed that PM correlated significantly with negative symptoms and duration of illness (DOI) in chronic schizophrenia [14]. Results in first episode patients have been more contradictory, with one study finding significant associations between PM and negative symptoms [16], while another one did not [17]. TBPM was found to be more impaired than EBPM [9], except in one study [18], which reported that the interaction between schizophrenia diagnosis and PM type (time-based versus event-based) was not significant. Some researchers suggested that PM deficits may be a primary rather than secondary deficit in schizophrenia [10], [12], [18]. However, this conclusion cannot be drawn with confidence, as it was not confirmed in all studies [16].

Schizophrenia has a strong heritable component [19] with heritability estimates of as high as 80% [20]. Cognitive impairment may be related to the genetic susceptibility for schizophrenia, as first-degree relatives (FDRs) show similar but less severe cognitive deficits compared to patients, including immediate and delayed recall, working memory, executive functions, spatial reasoning, verbal knowledge, perceptual and motor speed, attention and verbal fluency [21]–[30]. Research on PM in the population at high risk for schizophrenia is still in its infancy. To date, there have been only two studies addressing PM performance in FDRs of schizophrenia patients [17], [31]. While one study [31] found significant PM deficits in a small sample of parents and siblings (n = 26), a second study [17] did not observe a difference compared to HCs examining 40 nonpsychotic siblings of schizophrenia patients. Due to the small sample size and contradictory findings of the two prior studies, these findings require further examination.

The aims of the current study were (1) to determine whether schizophrenia patients’ first-degree relatives (including siblings and offspring) show impairment in TBPM and EBPM compared to healthy controls and to FES; and (2) to clarify the relationship between PM performance and demographic as well as clinical variables.

The first hypothesis was that FDRs would share similar but attenuated PM impairments with FES. The second hypothesis was that RM and executive functions would be significantly correlated with PM tasks in FDRs.

## Methods

### Participants

FDRs (23 offspring and 27 siblings) of schizophrenia patients were recruited as unaffected family members. Further, 47 first-episode schizophrenia patients from either outpatient or inpatient facilities in Beijing Anding Hospital (unrelated to the FDR group) were recruited. FES and FDRs were consecutively referred to the research team for screening of eligibility by their treating psychiatrists (for FES) or their psychotic relatives’ treating psychiatrist (for FDRs). A group of 51 healthy controls (HCs) matched with the FES group by age (±2 years) and education (±2 years) was recruited from the community through advertisements.

Familial high-risk individuals matched to the FES group by age (±2 years) and education (±2 years) and fulfilling the following study criteria were invited to participate in the study. (1) Offspring or siblings of patients with schizophrenia according to DSM-IV [32], [33]; (2) age between 16 and 45 years; (3) Chinese ethnicity; (4) at least primary level of education and having the ability to understand the requirements of the study; (5) willingness to give informed consent to take part in the study. Exclusion criteria were (1) history or current diagnosis of any psychiatric disorder; (2) history of or current major medical or neurological condition(s), including learning disability/mental retardation.

All patients with first-episode schizophrenia fulfilled the criteria of the Diagnostic and Statistical Manual of Mental Disorder-fourth edition (DSM-IV) [32] for schizophrenia established by administering the SCID-DSM-IV; [33] augmented by a review of medical records. In addition, they satisfied the following inclusion criteria (1) acute manifestations of the first episode of the illness; (2) age between 16 and 45 years; (3) at least primary level of education and the ability to understand the requirements of the study; (4) willingness to give informed consent to take part in the study. Exclusion criteria were: 1) history of, or current drug/alcohol abuse; and (2) history of, or current major medical or neurological condition(s), including learning disability and mental retardation.

HCs without history of or current psychiatric disorders, medical and neurological conditions were also recruited. The absence of psychiatric disorders was established with SCID-DSM-IV. The study protocol was approved by the Clinical Research Ethics Committee of Beijing Anding Hospital. Written informed consent was obtained from each participant.

### Assessment

#### Prospective memory

PM was assessed with the validated Chinese version of the Cambridge PM Test (C-CAMPROMPT) [34]. In this test, participants are asked to remember carrying out three TBPM and EBPM tasks each while working on a number of other activities (pencil and paper tasks, such as a general knowledge quiz or word-finding puzzle) for a 20 minute period. The C-CAMPROMPT generates scores on all six tasks, each with a maximum score of 6; higher scores indicate better PM performance.

Other neuropsychological assessments, targeting RM, verbal fluency, visual attention, and cognitive flexibility included:

1. *Hopkins Verbal Learning Test-revised, Chinese version* (HVLT-R; [35], to measure RM. The HVLT-R is a list-learning verbal memory test that has three learning trials (immediate recall) and a delayed recall. The total score of the immediate recall was used to measure RM.
2. *Verbal Fluency Test, Chinese version (VFT;* [35] to assess language and category fluency. VFT comprises four sections: two character (“phonemic”) tests and two category (sematic) tests. The separately averaged scores in the two character and two category trials yield an overall phrase performance and category verbal fluency performance, respectively.
3. *Color Trails Test (CTT).* The CTT [36], a culture-neutral version of the Trail Making Test (TMT; [37]), was selected to measure sustained visual attention. The CTT consists of two parts (CTT-1 and CTT-2). The CTT-1 requires participants to connect a series of numbered circles that are randomly printed on a sheet of paper. In the CTT-2, numbered circles of 1 to 25 are shown twice (printed in pink and in yellow) randomly on a sheet of paper. Participants are asked to connect the numbers from 1 to 25 alternating between the two colors.
4. *Stroop Color Word Test (SCWT), Chinese version;* [35] was administered to measure selective attention and cognitive flexibility. SCWT is composed of three parts, each lasting for 45 seconds. Only the score of the third part of the test - the Stroop Color-Word Interference - was used in this study.
5. *Wisconsin Card Sorting Test (WCST-64).* The computerized version of the WCST-64 was adopted to assess the prefrontal lobe function of reactive flexibility [38]. The number of categories completed (WCST-CC) and the percentage of perseverative errors (WCST-PE) were recorded.

### Procedures

All participants were tested in Beijing Anding Hospital. Neuropsychological tests were administered first in a randomized order to minimize the possibility of order effects. After a short break, the C-CAMPROMPT was administered.

The whole assessment lasted 1–2 hours and was completed on the same day for controls and relatives and within two consecutive days for patients, as some patients could not complete both tests and tasks on the same day.

All participants were asked to repeat the instructions before beginning the C-CAMPROMPT to ensure that they understood what was expected of them. The instructions were repeated and explained to participants who seemed uncertain about the nature of the test. Upon completion of the C-CAMPROMPT, participants were again asked to repeat the requirements of the test to make sure they remembered and understood the instructions. Two psychiatrists (FCZ and WMH) who were trained to assess PM and other cognitive functions administered the PM and other neurocognitive tests.

### Data analysis

The data were analyzed using SPSS, Version 20.0 for Windows. Comparisons between FDRs, FES and HCs with regard to socio-demographic characteristics and mean scores of all neurocognitive tests were performed using independent sample one-way ANOVA, ANCOVA, Mann-Whitney U tests and chi-square tests, as appropriate. Comparisons between siblings and offspring regarding TBPM and EBPM were performed using independent-samples t-tests. Pearson’s or Spearman rank correlation analysis was performed to determine the associations between performance on TBPM and EBPM tasks and socio-demographic and other neurocognitive tests. Factors independently associated with the performance on the TBPM and EBPM in FDRs were identified with stepwise multiple linear regression analyses. The normality of distributions for the continuous variables was checked with the one-sample Kolmogorov-Smirnov test. Two-tailed tests were used in all analyses with the significance level set at 0.05.

## Results

Altogether, 148 subjects were included in this study, i.e., 47 FES patients, 50 relatives of chronic schizophrenia patients and 51 controls. Subjects were on average in their mid to end 20s and 43–60% were male. Table 1 presents the basic demographic variables, the cognitive test results in the three groups, and the clinical characteristics of the FES group. There was a significant difference between the three groups in education. Table 2 shows the post-hoc analyses of TBPM and EBPM. After controlling for basic demographic characteristics including age, gender and educational level by analysis of covariance (ANCOVA), the difference between the three groups remained significant with respect to both TBPM (F_(2,142)_ = 10.4, p<0.001) and EBPM (F_(2,142)_  = 10.8, p<0.001). In addition, we compared TBPM and EBPM between the siblings and offspring and did not find any significant difference between these two subgroups (Table 3).

**Table 1:** Comparison of first episode schizophrenia patients, first-degree relatives of patients with chronic schizophrenia and healthy controls with respect to demographic, neurocognitive and clinical characteristics.

	Patients (n = 47)		Relatives (n = 50)		Controls (n = 51)		Statistics	
Variable	N	Percent	N	Percent	N	Percent	*x* ^2^	*p*-value
Men	28	59.6	26	52.0	18	42.9	2.974	0.226
On antipsychotics	24	51.1	–	–	–	–	–	–
On anticholinergics	10	21.3	–	–	–	–	–	–
	**Mean**	**SD**	**Mean**	**SD**	**Mean**	**SD**	***F/x*** **^2^**	***p*** **-value**
Age (year)	25.5	6.5	28.0	6.3	24.3	4.7	4.8	0.1
Education (year)	14.1	1.8	14.0	2.7	16.1	2.6	16.8^k^	<0.001
Age at onset (year)	24.4	5.9	–	–	–	–	–	–
DUP (month)	12.8	11.7	–	–	–	–	–	–
PANSS positive	25.3	4.3	–	–	–	–	–	–
PANSS negative	22.5	6.8	–	–	–	–	–	–
PSP	41.3	9.8	–	–	–	–	–	–
TBPM	9.1	5.3	11.4	4.0	14.8	3.5	33.5 ^k^	<0.001
EBPM	11.5	4.7	13.8	3.2	15.7	2.7	27.6^k^	<0.001
HVLT-R	22.0	6.8	26.2	5.2	29.5	4.4	30.6 ^k^	<0.001
CTT-1	59.0	14.5	38.7	11.0	46.3	14.3	32.3	<0.001
CTT-2	93.0	21.3	80.7	23.3	76.6	19.7	8.9	<0.001
SCWT	33.8	9.4	37.3	11.5	46.6	11.0	31.8 ^k^	<0.001
WCST-PE	11.3	7.5	18.7	6.6	7.2	3.8	64.5 ^k^	<0.001
WCST-CC	2.9	1.6	2.4	1.8	3.4	1.4	10.8 ^k^	0.009
VFL	2.7	0.9	3.0	1.3	3.8	1.6	15.5 ^k^	<0.001
VFC	13.2	3.7	14.8	3.3	17.9	3.4	23.9	<0.001

CTT = Color Trails Test; DUP = Duration of untreated psychosis; EBPM = Event-based prospective memory; HVLT-R = Hopkins Verbal Learning Test-Revised Version; k = Kruskal-Wallis H test; PANSS = Positive and Negative Syndrome Scale; PSP = Personal and Social Performance Scale; SCWT = Stroop Color Word Test; WCST-PE = Wisconsin Card Sorting Test (percentage of perseverative errors); WCST-CC = Wisconsin Card Sorting Test (number of categories completed); TBPM = Time-based prospective memory; VFC = Verbal Fluency Test (Category test); VFL = Verbal Fluency Test (Letter test).

**Table 2:** Post-hoc analyses of TBPM and EBPM.

Prospective Memory Task	(I) Group	(II) Group	Mean Difference (I–II)	p-value
TBPM	Relatives (n = 50)	Patients (n = 47)	2.2	0.02
		Controls (n = 51)	−2.7	0.003
EBPM	Relatives (n = 50)	Patients (n = 47)	2.3	0.003
		Controls (n = 51)	−1.5	0.038

EBPM = Event-based prospective memory; TBPM = Time-based prospective memory.

**Table 3:** Comparison of siblings and offspring with respect to TBPM and EBPM.

Prospective Memory Task	Siblings (n = 27)		Offspring (n = 23)		Statistics	
	Mean	SD	Mean	SD	*t*	*p*-value
TBPM	11.3	4.8	11.5	3.0	−0.2	0.84
EBPM	14.2	3.2	13.4	3.2	1.0	0.33

EBPM = Event-based prospective memory; TBPM = Time-based prospective memory.

Table 4 presents the relationships between PM and socio-demographic variables, other cognitive tests, and clinical variables. For FDRs, lower scores on the HVLT-R, and SCWT, and higher scores of CTT-1 and CTT-2 were significantly associated with poorer TBPM performance. Conversely, female gender, lower educational level, lower scores of HVLT-R and VFC, and higher scores of the CTT-2 were associated with poorer EBPM performance.

**Table 4:** Correlations between prospective memory tasks and demographic and clinical characteristics in schizophrenia patients, first-degree relatives and healthy controls.

	Patients (n = 47)		Relatives (n = 50)		Controls (n = 51)		
Variable	TBPM	EBPM	TBPM	EBPM	TBPM	EBPM	
Age (year)	−0.22	−0.19	−0.002	0.14	0.02	0.01	
Gender	−0.16	−0.04	0.23	0.35*	−0.03	−0.14	
Education (year)	0.09	−0.24	0.26	0.33*	0.09	0.07	
Age at onset (year)	−0.17	−0.15	–	–	–	–	
DUP (month)	−.42**	−0.29*	–	–	–	–	
PANSS positive	−0.12	−0.09	–	–	–	–	
PANSS negative	−.44**	−0.40**	–	–	–	–	
On antipsychotics	−0.16	−0.06	–	–	–	–	
On anticholinergics	−0.07	−0.28	–	–	–	–	
HVLT-R	0.46**	0.46**	0.39**	0.28*	−0.03	0.13	
CTT-1	−0.30*	−0.11	−0.29*	−0.24	−0.2	−0.23	
CTT-2	−0.42**	−0.23	−0.40**	−0.34*	−0.06	0.09	
SCWT	0.28	0.36*	0.49**	0.23	0.05	−0.13	
WCST-PE	−0.36*	−0.47**	−0.24	−0.09	0.02	0.10	
WCST-CC	0.42**	0.45**	0.29*	0.09	0.05	−0.13	
VFL	0.10	0.23	0.07	0.14	0.02	0.07	
VFC	0.30*	0.36*	0.20	0.28*	0.2	0.05	

*p<0.05;

**p<0.01;

CTT = Color Trails Test; DUP = Duration of untreated psychosis; EBPM = Event-based prospective memory; HVLT-R = Hopkins Verbal Learning Test-Revised Version; PANSS = Positive and Negative Syndrome Scale; SCWT = Stroop Color Word Test; TBPM = Time-based prospective memory; VFC = Verbal Fluency Test (Category test); VFL = Verbal Fluency Test (Letter test); WCST-CC = Wisconsin Card Sorting Test (number of categories completed); WCST-PE = Wisconsin Card Sorting Test (percentage of perseverative errors).

Table 5 shows the independent correlates of TBPM and EBPM performance in FDRs. In the regression analyses, TBPM and EBPM scores were entered as the dependent variable separately, while all variables that showed significant correlations with PM tasks were entered as independent variables. Lower scores of HVLT-R and SWCT (total R^2^ = 0.30, p<0.001) independently contributed to poorer TBPM performance, while lower educational level, and higher scores of CTT-2 contributed to worse EBPM performance (total R^2^ = 0.17, p = 0.005).

**Table 5:** Results of the stepwise multiple regression analysis (First-degree relatives; n = 50).

Prospective Memory task	Predictor	Beta	p-value	95% CI
TBPM*	SCWT	0.4	0.001	0.06–0.23
	HVLT-R	0.3	0.02	0.03–0.4
EBPM#	CTT-2	−0.3	0.02	−0.07–−0.006
	Education	0.3	0.03	0.03–0.6

*Adjusted R^2^ = 0.30; F_(2,47)_ = 11.6; p<0.001;

# Adjusted R^2^ = 0.17; F_(2,47)_ = 6.0; p = 0.005;

CTT = Color Trails Test; DUP = Duration of untreated psychosis; EBPM = Event-based prospective memory; HVLT-R = Hopkins Verbal Learning Test-Revised Version; TBPM = Time-based prospective memory; WCST-CC = Wisconsin Card Sorting Test (number of categories completed); WCST-PE = Wisconsin Card Sorting Test (percentage of perseverative errors).

## Discussion

Confirming our first hypothesis, FDRs performed significantly poorer on both TBPM and EBPM compared to HCs, and shared a similar pattern of PM performance with schizophrenia patients [9], [11], [12], [39] before and after controlling for potential confounding effects of basic demographic characteristics. This finding suggests that PM may be related to the genetic susceptibility for schizophrenia and that it could be considered as a potential endophenotype. In addition, PM impairment is often regarded as a primary deficit in schizophrenia [17], [18], [40], [41]. The results in FDRs partly support this notion because after controlling for potentially confounding demographic variables, the difference in PM performance between FDRs, FES and HCs remained significant.

Previous studies found that FDRs of schizophrenia patients are a heterogeneous group regarding their risk for developing schizophrenia. The risk in offspring is approximately 13%, much higher than that in schizophrenia patients’ parents (6%) and siblings (9%) [42]. Offspring have been reported to have worse performance on neuropsychological tests than siblings [43]. Based on the abovementioned evidence, we assumed that offspring would have worse PM performance compared to siblings. However, our result did not support this assumption; yet the number of people in each subgroups was relatively small. Therefore, whether PM performance varies across the heterogeneous subgroups of relatives requires further study.

Neurocognitive impairments have been observed in both siblings [27], [44] and offspring [44], [45], [46] of patients with schizophrenia compared to HCs. Among cognitive tasks and domains, low intelligence is one of the most frequently reported areas. Earlier studies consistently found that high-risk offspring had lower IQ scores than HCs [26], [47], [48], [49]. In addition, low IQ was observed in unaffected siblings of schizophrenia patients [27]. In our study, those with intellectual problems were excluded, thus the impact of low IQ on PM could not be explored.

Neuroimaging studies have detected prefrontal and temporal grey matter deficits in monozygotic twins discordant for schizophrenia, siblings and other close relatives of participants with schizophrenia; and these deficits may be even progressive [50], [51]. The grey matter loss mainly involves the temporal lobes and poles, inferior frontal lobes, insula, medial and more lateral frontal regions, as well as to a lesser extent most of the cortical gray matter [52], [53]. These findings indicate that the decrease in prefrontal and temporal grey matter is heritable in schizophrenia, and, as such, could be regarded as an endophenotype of the illness. However, most previous studies employed tests designed at measuring a single cognitive function [52], [53], which did not reflect the interaction of two or more brain areas or the functional connectivity of the brain. There is a growing body of evidence showing that the functional connectivity between the prefrontal and temporal cortices is also impaired in persons at familial risk for schizophrenia [54]. Functional connectivity between the prefrontal and temporal cortices is also impaired in persons at familial risk for schizophrenia [54]. Electrophysiological studies indicated disturbed left frontotemporal interaction in siblings of schizophrenia patients [55]. Based on the findings summarized above, we speculate that the structural abnormalities and functional dysconnectivity in the frontotemporal circuits might be responsible for the PM deficit in persons with genetic risk for schizophrenia.

Regression analyses for the FDRs group showed that HVLT scores independently contributed to TBPM in FDRs. This may be related to the fact that PM involves a retrospective component [7], which retrieves the intended content and the specific action that must be executed [8]. The RM component of PM has been described to depend on the temporal cortex. In PET and fMRI studies, the left parahippocampal and the middle temporal gyri were activated by PM cues during PM tasks, which was thought to play a role in recognition of cues triggering the performance of intended actions [56], [57], [58], [59]. As expected, higher educational levels in FDRs was also significantly correlated with better PM performance, similar to findings in schizophrenia patients [11].

In this study, multivariate analyses revealed that prefrontal dysfunction plays a key role in the PM impairment in FDRs of schizophrenia patients. This finding is supported by neuroimaging studies. Increased activity during PM tasks is seen in the anterior and lateral prefrontal cortex, while decreased activity is observed in the medial prefrontal cortex using functional MRI [56], [60], [61], [62].

Regression analyses also indicated that although PM deficits could be attributed to dysfunctions in the frontal cortex in FDRs, TBPM and EBPM may depend on different neurocognitive processes. While TBPM was predicted by SCWT, EBPM was predicted by CTT-2. Brain imaging studies have shown that the anterior cingulate cortex and the dorsolateral prefrontal cortex are activated by the Stroop test. Specifically, while the left dorsolateral prefrontal cortex activation is related to the expectation regarding the conflicting nature of the upcoming trial, the right dorsolateral prefrontal cortex is activated when attempting to reduce the attentional conflict [63]. The anterior cingulate cortex is involved in selecting an appropriate response and allocate attentional resources, while the posterior dorsolateral prefrontal cortex is employed in accomplishing the current goal [64]. In the CTT-2, participants have to divide their attention while simultaneously tracking the alternating sequence of colors and numbers [65]. This process is related to the lower third of the dorsolateral prefrontal, premotor and left medial frontal cortex, and the intraparietal sulcus bilaterally [66].

The results of our regression analysis, together with the aforementioned line of evidence, suggest that the prefrontal and temporal cortices may act as an integrated circuit during PM tasks in FDRs of schizophrenia. This hypothesis seems to be supported by a previous study, which showed that the intermediate CA1 region of the hippocampus and the medial prefrontal cortex interact in coordinating RM and PM processes in anticipation of obtaining a remote goal [67]. Earlier neurocognitive studies suggested that dopaminergic modulation of hippocampal-prefrontal cortical interaction plays a key role in PM. In one study [68], RM appeared to depend on D1 receptor function, which selects the information from the hippocampus to be incorporated into the prefrontal cortex network. This information is further processed by D2 receptor activity within the prefrontal cortex. Functional dysconnectivity between the prefrontal cortex and limbic structures, and dopaminergic modulation of that dysconnectivity have been described in schizophrenia patients and their first-degree relatives [69], [70], [71], which could partly explain their PM deficits.

The strengths of this study include the relatively large sample size that comprised all types of FDRs and pertinent cognitive assessment battery. However, due to methodological limitations, the results should be interpreted with caution. First, controls and relatives were matched with patients separately in terms of age and education, but the three groups still had significant differences in education level. Nevertheless, findings remained significant even after controlling for the potentially confounding effect of educational level. Second, in multivariate analyses the significant variables only accounted for 30.2% and 17.0% of the variance in TBPM and EBPM, respectively. In order to explore the impact of more variables on PM, a larger sample size will be required in future studies. Third, the cross-sectional design could not explore causal relationships between PM and other variables. Fourth, analyses comparing siblings and offspring of schizophrenia patients were limited by the small sample size of these subgroups.

In conclusion, the major findings of this study are: (1) FDRs of schizophrenia patients showed significant impairment in PM; (2) RM deficits and neurocognitive markers of prefrontal dysfunction independently correlated with PM impairment; and (3) both FES and FDRs of schizophrenia showed PM impairment suggesting that PM may be an endophenotype for schizophrenia.
